# SILEN-C3, a Phase 2 Randomized Trial with Faldaprevir plus Pegylated Interferon α-2a and Ribavirin in Treatment-Naive Hepatitis C Virus Genotype 1-Infected Patients

**DOI:** 10.1128/AAC.02497-13

**Published:** 2014-06

**Authors:** Douglas Dieterich, Tarik Asselah, Dominique Guyader, Thomas Berg, Marcus Schuchmann, Stefan Mauss, Vlad Ratziu, Peter Ferenci, Dominique Larrey, Andreas Maieron, Jerry O. Stern, Melek Ozan, Yakov Datsenko, Wulf Otto Böcher, Gerhard Steinmann

**Affiliations:** a Division of Liver Diseases, Icahn School of Medicine at Mount Sinai, New York, New York, USA; b Service d'Hépatologie, AP-HP Hôpital Beaujon, INSERM UMR 1149, CRI, Université Paris Diderot, Clichy, France; c Department of Liver Diseases, University of Rennes 1, Rennes, France; d Sektion Hepatologie, Department für Innere Medizin, Neurologie und Dermatologie, Klinik für Gastroenterologie und Rheumatologie, Universitätsklinik Leipzig AöR, Leipzig, Germany; e Klinik für Innere Medizin, Klinikum Konzstanz, Constance, Germany; f Center for HIV and Hepatogastroenterology, Düsseldorf, Germany; g Service d'Hépato-Gastroentérologie, Hôpital Pitié-Salpêtrière, Paris, France; h Department of Internal Medicine III, Division of Gastroenterology and Hepatology, Medical University of Vienna, Vienna, Austria; i Liver Unit-IRB-INSERM1040, Hôpital Saint Eloi, Montpellier, France; j Department of Gastroenterology and Hepatology, Elisabethinen Hospital Linz, Linz, Austria; k Boehringer Ingelheim Pharmaceuticals Inc., Ridgefield, Connecticut, USA; l Boehringer Ingelheim Pharma GmbH & Co. KG, Biberach, Germany; m Boehringer Ingelheim Pharma GmbH & Co. KG, Ingelheim, Germany

## Abstract

Faldaprevir is an investigational hepatitis C virus (HCV) NS3/4A protease inhibitor which, when administered for 24 weeks in combination with pegylated interferon α-2a and ribavirin (PegIFN/RBV) in treatment-naive patients in a prior study (SILEN-C1; M. S. Sulkowski et al., Hepatology 57:2143–2154, 2013, doi:10.1002/hep.26276), achieved sustained virologic response (SVR) rates of 72 to 84%. The current randomized, open-label, parallel-group study compared the efficacy and safety of 12 versus 24 weeks of 120 mg faldaprevir administered once daily, combined with 24 or 48 weeks of PegIFN/RBV, in 160 treatment-naive HCV genotype 1 patients. Patients with maintained rapid virologic response (HCV RNA of <25 IU/ml at week 4 and undetectable at weeks 8 and 12) stopped all treatment at week 24, otherwise they continued PegIFN/RBV to week 48. SVR was achieved by 67% and 74% of patients in the 12-week and 24-week groups, respectively. Virologic response rates were lower in the 12-week group from weeks 2 to 12, during which both groups received identical treatment. SVR rates were similar in both groups for patients achieving undetectable HCV RNA. Most adverse events were mild or moderate, and 6% of patients in each treatment group discontinued treatment due to adverse events. Once-daily faldaprevir at 120 mg for 12 or 24 weeks with PegIFN/RBV resulted in high SVR rates, and the regimen was well tolerated. Differences in the overall SVR rates between the 12-week and 24-week groups were not statistically significant and possibly were due to *IL28B* genotype imbalances; *IL28B* genotype was not tested, as its significance was not known at the time of the study. These results supported phase 3 evaluation. (This study has been registered at ClinicalTrials.gov under registration no. NCT00984620).

## INTRODUCTION

Chronic hepatitis C virus (HCV) infection is a major health problem worldwide, with patients at risk of progressing to liver cirrhosis and hepatocellular carcinoma (1, 2). The NS3/4A protease inhibitors boceprevir and telaprevir were a major advance in the treatment of chronic HCV genotype 1 (GT-1) infection (3, 4). Addition of boceprevir or telaprevir to pegylated interferon α-2a (PegIFN) and ribavirin (RBV) increased sustained virologic response (SVR) rates compared to those of the placebo in HCV GT-1-infected patients (5–7) and enabled 40 to 60% of treatment-naive patients to reduce the treatment duration to 24 or 36 weeks rather than the 48 weeks required with PegIFN/RBV alone (3, 5). Shortening treatment duration is desirable in order to reduce the side effects associated with PegIFN and RBV.

However, boceprevir and telaprevir are associated with serious side effects, including rash and anemia, carry a high pill burden, require dosing every 8 h, and have numerous drug-drug interactions (3–7). Their use in clinical practice in patients with cirrhosis (8, 9) showed substantially higher mortality rates and an increased prevalence of severe side effects compared to clinical trials (5, 6, 10, 11). New direct-acting antivirals with improved tolerability, convenience, and drug-drug interaction profiles are needed (12).

Faldaprevir is an effective and highly specific noncovalently binding, linear HCV NS3/4A protease inhibitor with a pharmacokinetic profile conducive to once-daily (QD) dosing (13). In phase 1b studies, faldaprevir plus PegIFN/RBV induced profound antiviral responses in HCV GT-1 treatment-naive and treatment-experienced patients (13). The principal phase 2 program of faldaprevir consisted of the SILEN-C1 (in treatment-naive patients) and SILEN-C2 (in treatment-experienced patients) studies (NCT00774397). SILEN-C1, a large, double-blind, placebo-controlled study, showed that faldaprevir (120 or 240 mg QD for 24 weeks) plus PegIFN/RBV achieved higher SVR rates (72 to 84%) than PegIFN/RBV alone (56%) (14). SILEN-C3 (NCT00605098), initiated while SILEN-C1 and SILEN-C2 were still in progress, was added to the phase 2 program to address unanswered questions regarding optimal treatment duration with faldaprevir raised after the publication of data from other protease inhibitors (7, 15). The objective virologic endpoints of SILEN-C3 allowed the use of an open-label and uncontrolled study design. The 120-mg dose of faldaprevir was selected for SILEN-C3 based on the results of a 4-week study showing that the 120-mg and 240-mg faldaprevir doses had similar antiviral activity (13). At the time SILEN-C3 was designed, a lead-in period, in which patients received PegIFN/RBV alone for the first 3 days of therapy, was included in both treatment arms. This was based on the hypothesis that achieving sufficient plasma levels of PegIFN/RBV would avoid functional faldaprevir monotherapy and could minimize the early emergence of resistance mutations. Here, we report the results of the SILEN-C3 trial, comparing the efficacy and safety of 12 versus 24 weeks of faldaprevir at 120 mg QD plus PegIFN/RBV in treatment-naive HCV GT-1-infected patients. The study included patients with compensated liver cirrhosis.

## MATERIALS AND METHODS

### Patients.

Eligible patients were aged 18 to 70 years, naive to interferon, PegIFN, and RBV, and had chronic HCV GT-1 infection (positive HCV serology for >6 months or liver histology typical of chronic hepatitis plus HCV RNA of ≥100,000 IU/ml at screening). Patients had a liver biopsy or Fibroscan within 2 years of screening to assess fibrosis or cirrhosis. A normal retinal fundoscopy within 6 months of study day 1 was required. Key exclusion criteria included liver disease resulting from causes other than chronic HCV, HCV of mixed genotype, hepatitis B virus infection, HIV infection, decompensated liver disease, or total bilirubin of >1.5× the upper limit of normal (ULN).

### Study design.

**FIG 1:**
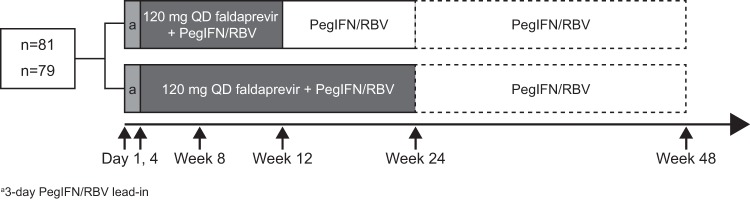
Study design. Treatment-naive patients were randomized to 12 or 24 weeks of faldaprevir plus pegylated interferon α-2a (PegIFN) and ribavirin (RBV), followed by PegIFN/RBV alone. Patients who achieved a maintained rapid virologic response (mRVR; HCV RNA below the lower limit of quantification [LLOQ; <25 IU/ml] at week 4 and undetected at weeks 8 and 12) stopped all treatment at week 24; those who did not achieve mRVR continued PegIFN/RBV to week 48. QD, once daily.

Patients were recruited by specialists experienced in treating HCV and were screened and enrolled at 27 centers in Austria, Canada, France, Germany, Romania, and the United States. Patients were randomized 1:1 to faldaprevir at 120 mg QD for 12 or 24 weeks plus PegIFN/RBV for 24 or 48 weeks (Fig. 1). A dose of 120 mg was chosen for this study, because previous trials had shown it to have similar efficacy and better tolerability than a 240-mg dose (14). Patients who achieved a maintained rapid virologic response (mRVR; HCV RNA below the lower limit of quantification [LLOQ; <25 IU/ml] at week 4 and undetectable at weeks 8 and 12) stopped all treatment at week 24; those who did not achieve mRVR continued PegIFN/RBV to week 48.

A 3-day lead-in of PegIFN/RBV was used in both treatment arms. This was intended to allow patients to achieve sufficient plasma and hepatic levels of PegIFN/RBV before the first administration of faldaprevir, thereby avoiding a period of early functional monotherapy and minimizing the risk of selecting resistant virus. Patients received a 240-mg loading dose of faldaprevir at the first administration followed by 120 mg QD thereafter. The loading dose allowed patients to achieve steady state earlier. PegIFN (F. Hoffmann-La Roche Ltd., Basel, Switzerland), 180 μg per week, was administered subcutaneously. RBV (F. Hoffmann-La Roche Ltd., Basel, Switzerland), 1,000 mg (body weight of <75 kg) or 1,200 mg (body weight of ≥75 kg) per day, was given orally.

The stopping rules were the following: HCV RNA load of ≥1,000 IU/ml at two consecutive visits at least 2 weeks apart after previously undetected HCV RNA, lack of early virologic response (absence of HCV RNA reduction by ≥2 log_10_ from baseline at week 12), or detectable HCV RNA at week 24.

Visits were scheduled for all patients for weeks −4 to −1 (screening), day 1, and for weeks 2, 4, 8, 12, 18, and 24. Patients who achieved mRVR stopped all treatment at week 24 and had follow-up visits at weeks 28, 36, and 48 (4, 12, and 24 weeks after the end of treatment [EOT]). Patients who did not achieve mRVR continued PegIFN/RBV through week 48 and had follow-up visits at weeks 60 and 72 (12 and 24 weeks after EOT). For patients who discontinued early, a follow-up visit was scheduled within 7 days of the last dose of study drug.

All patients followed a rash management plan. The plan included definitions of the severity of treatment-emergent rash and other skin reactions, the use of sunscreen by all patients, and protocols for management of skin reactions (including immediate discontinuation of all study medications in the event of severe rash).

The randomization list was generated by a pseudo-random number generator, with a third-party interactive voice response system (IVRS) to assign randomization numbers. All patients provided written informed consent, and the study was conducted in accordance with the principles of the Declaration of Helsinki, followed the International Conference on Harmonised Tripartite Guideline for Good Clinical Practice, and was registered at www.clinicaltrials.gov (NCT00984620). Study materials were approved by independent ethics committees or institutional review boards of participating centers and by the relevant authorities in each country.

### Study endpoints.

The primary endpoint was virologic response (HCV RNA undetected) at week 28 (termed the week 28 virologic response, W28VR) and was selected to inform the phase 3 dose regimen selection. Key secondary endpoints included SVR (HCV RNA undetectable) 24 weeks after completion of all therapy, rapid virologic response (RVR; HCV RNA undetectable at week 4), time to reach undetectable HCV RNA levels in plasma, safety, and tolerability. Patients without data 24 weeks after the end of therapy were recorded as SVR failures.

### Efficacy assessments.

Plasma HCV RNA levels were measured using the COBAS TaqMan HCV/HPS assay (Roche Molecular Diagnostics) at a central laboratory (Covance Central Laboratory Services). The LLOQ was 25 IU/ml. Viral genotyping included population sequencing of the NS3/NS4A region. Genotyping was performed for all patients at baseline, for patients who discontinued study treatment, and on samples from patients whose HCV RNA levels reached a plateau above the LLOQ or rebounded during the study period.

### Safety assessments.

All adverse events (AEs), including time of onset, end time, intensity, intervention, and outcome, were reported in writing by the investigator to the sponsor based on a patient's toleration of the event as mild (easy to tolerate), moderate (interference with usual activity), or severe (incapacitating or preventing work or usual activities). Rash was graded by the investigator as mild (localized), moderate (diffuse, 30 to 70% body surface area), or severe (diffuse, generalized, >70% body surface area, mucous membranes involved, organ dysfunction, signs of anaphylaxis, or life threatening). Vital signs, electrocardiogram, and routine laboratory parameters were evaluated. Growth factors (e.g., erythropoiesis-stimulating agents and granulocyte colony-stimulating factor) were permitted at the investigator's discretion in the event of anemia or neutropenia.

### Statistical assessments.

The sample size was adopted from the SILEN-C1 trial (14) and was calculated from the expected response rates and the effect of sample size on the likelihood of false-positive or false-negative results. This showed that 70 patients in each treatment arm would be appropriate. No formal statistical testing was planned, and no hypotheses were defined. Descriptive statistics for efficacy and safety were presented. For efficacy endpoints, to provide a quantitative assessment of the magnitude of differences in efficacy between the two durations, 95% confidence intervals (CI) along with corresponding 2-sided *P* values were calculated using Cochran-Mantel-Haenszel methods adjusted for GT-1 subtype (1a or 1b). However, since the study was not designed or powered to allow any formal testing of differences between the two durations, these should be interpreted only descriptively without inference being made to a larger population of patients.

## RESULTS

### Patient disposition and baseline characteristics.

**FIG 2:**
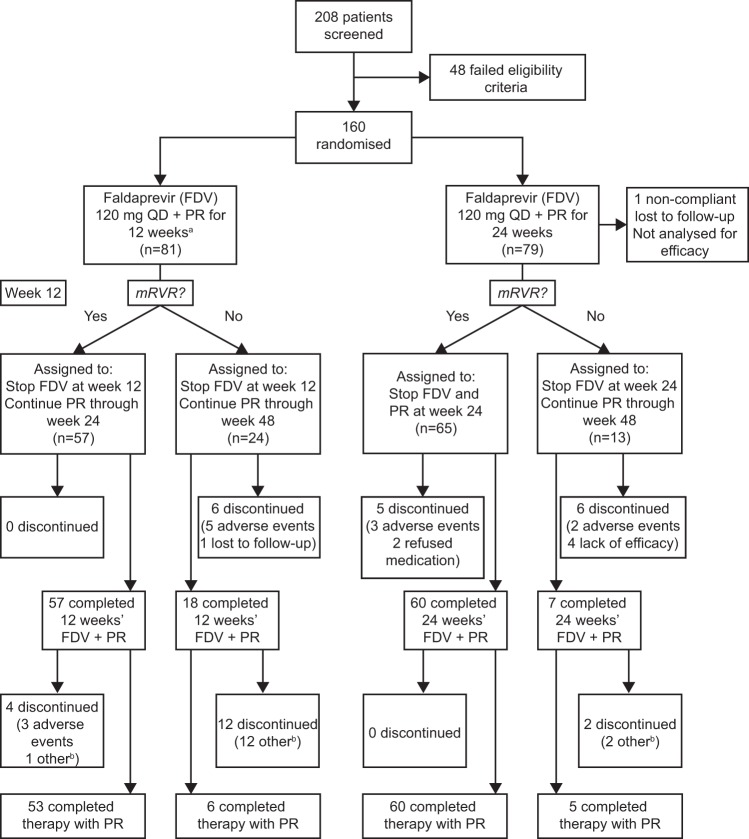
Patient disposition. Footnotes: a, one patient experienced severe bone pain during PR lead-in and discontinued before receiving FDV; b, includes lack of efficacy and lost to follow-up. QD, once daily; PR, pegylated interferon α-2a and ribavirin; mRVR, maintained rapid virologic response (HCV RNA below the lower limit of quantification [LLOQ; <25 IU/ml] at week 4 and undetectable at weeks 8 and 12).

Between September and October 2009, 208 patients were enrolled, and 160 entered into the trial; 81 were randomized to the 12-week group and 79 to the 24-week group (Fig. 2). All randomized patients received at least one dose of study drug. One patient in the 24-week group discontinued faldaprevir on day 82 and PegIFN/RBV on day 85 and then was lost to follow-up. This was deemed to be a major protocol violation, and the patient was excluded from efficacy analysis according to the protocol. Premature discontinuation for any reason occurred in 7% (6/81) of patients in the 12-week group and 15% (12/79) in the 24-week group during the treatment phase; five patients (6%) in each group discontinued because of AEs. Of the five patients in the 12-week group, one discontinued faldaprevir and PegIFN/RBV due to the diagnosis of prostate cancer during screening. Another discontinued PegIFN/RBV during lead-in due to an AE (bone pain) and did not receive faldaprevir.

Ethnicity, body mass index, and presence of liver cirrhosis were evenly balanced in the treatment groups, but the 12-week group was older and had more patients with HCV RNA of ≥800,000 IU/ml and GT-1a infection (Table 1). *IL28B* testing was not performed in this study, since its predictive effect on response to IFN-based treatment had not yet been established when the study was initiated.

**TABLE 1:** Baseline characteristics

Characteristic	Value by faldaprevir treatment duration	
	12 wk (*n* = 81)	24 wk (*n* = 79)
Mean age, yrs (SD)	48.1 (9.4)	44.9 (11.9)
Male, *n* (%)	54 (67)	45 (57)
Body mass index, kg/m^2^, means (SD)	25.3 (4.5)	25.8 (4.1)
Race, *n* (%)		
White	77 (95)	76 (96)
Asian	2 (3)	2 (3)
Black	2 (3)	1 (1)
Log_10_ HCV RNA, means (SD)	6.53 (0.49)	6.46 (0.61)
HCV RNA level, *n* (%)		
<800,000 IU/ml	9 (11)	17 (22)
≥800,000 IU/ml	72 (89)	62 (79)
HCV genotype,^*a*^ *n* (%)		
1a	40 (49)	29 (37)
1b	39 (48)	49 (62)
1g	1 (1)	1 (1)
6e	1 (1)	0
Liver cirrhosis,^*b*^ *n* (%)		
Yes	10 (12)	10 (13)
No	71 (88)	69 (87)

a Based on NS3/4A sequencing.

b Reported by the investigator.

### Efficacy.

**FIG 3:**
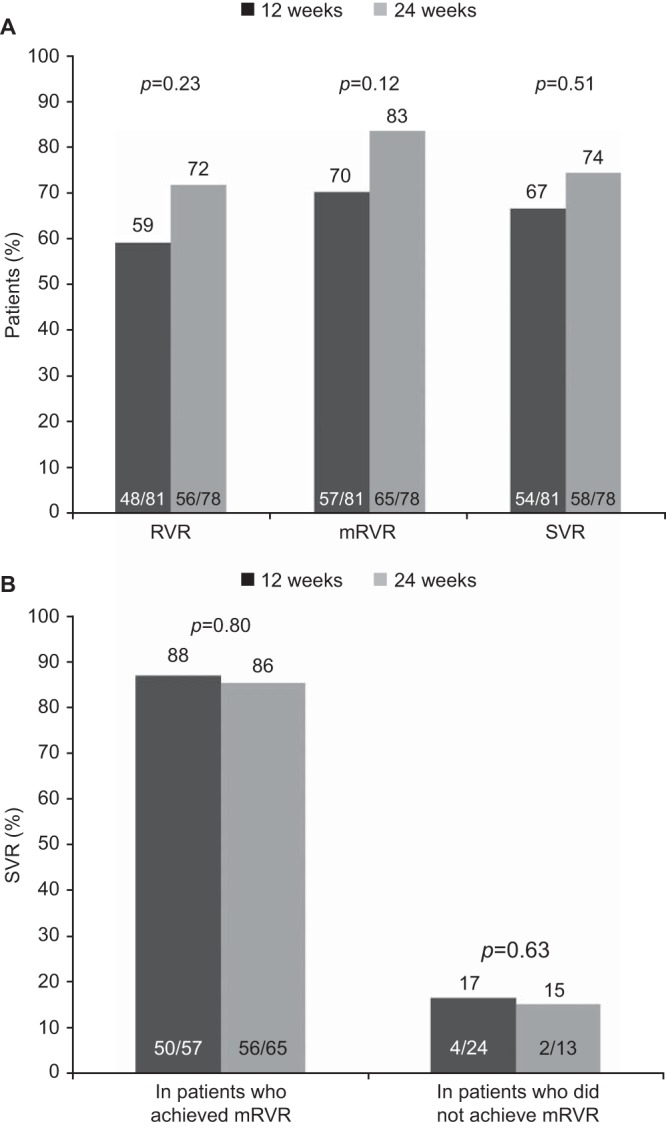
(A) Rapid virologic response (RVR), maintained rapid virologic response (mRVR), and sustained virologic response (SVR) rates by treatment group. (B) SVR rates in patients who achieved mRVR versus those who did not achieve mRVR. *P* values (two sided) were calculated using the Cochran-Mantel-Haenszel test, adjusted for genotype 1 subtype (1a or 1b). RVR, HCV RNA undetectable at week 4; mRVR, HCV RNA below the lower limit of quantification [LLOQ; <25 IU/ml] at week 4 and undetectable at weeks 8 and 12); SVR, HCV RNA undetectable at 24 weeks posttreatment.

**FIG 4:**
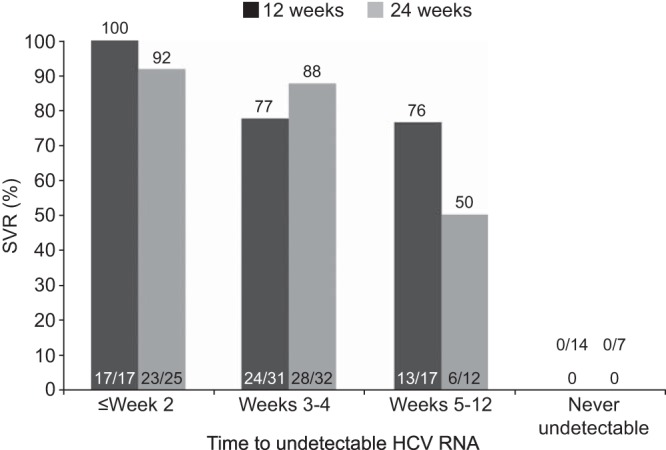
Sustained virologic response (SVR) rates in relation to time taken to achieve undetectable HCV RNA. A shorter time to achieve undetectable HCV RNA was associated with higher likelihood of SVR.

The SVR rate was 67% (54/81) in the 12-week group and 74% (58/78) in the 24-week group (adjusted difference, 4.78%; 95% CI, 9.0, 18.6; *P* = 0.51) (Table 2). Of the 10 patients in each treatment group with compensated liver cirrhosis at baseline, 3 patients in the 12-week group and 4 in the 24-week group achieved SVR. RVR was achieved by 59% (48/81) and 72% (56/78) of patients in the 12-week and 24-week groups, respectively (adjusted difference, 9.02%; 95% CI, −5.3, 23.3; *P* = 0.23). The primary endpoint (W28VR) was met by 75% (61/81) and 77% (60/78) of patients in the 12- and 24-week groups, respectively (adjusted difference, −1.25%; 95% CI, −14.3, 11.8; *P* = 0.86). In each treatment group, 60% (6/10) of patients with compensated liver cirrhosis at baseline achieved W28VR. The criterion for shortened overall treatment duration, mRVR, was achieved by 70% (57/81) and 83% (65/78) of patients in the 12- and 24-week groups, respectively (adjusted difference, 10.36%; 95% CI, −1.9, 22.6; *P* = 0.12); these patients stopped all treatment at week 24 (Fig. 3A). Their SVR rates were 88% (50/57) and 86% (56/65), respectively. Patients without mRVR were assigned by protocol to receive 48 weeks of treatment and had SVR rates of <20% (Fig. 3B). Shorter time to achieve undetectable HCV RNA was associated with higher likelihood of SVR; 95% (40/42) of patients with HCV RNA undetectable by week 2 achieved SVR (Fig. 4).

**TABLE 2:** Virologic response

Response category^*d*^	Value (no. [%]) by faldaprevir treatment duration		Adjusted difference (95% CI)
	12 wk^*a*^ (*n* = 81)	24 wk^*b*^ (*n* = 78)	
W28VR	61 (75.3)	60 (76.9)	−1.25 (−14.3, 11.8)
RVR	48 (59.3)	56 (71.8)	9.02 (−5.3, 23.3)
SVR12	56 (69.1)	59 (75.6)	3.41 (−10.1, 16.9)
SVR24^*c*^	54 (66.7)	58 (74.4)	4.78 (−9.0, 18.6)

a Includes one patient with NS3/NS4 GT-6e (achieved SVR12 and SVR24).

b One patient was noncompliant and lost to follow-up and was excluded from efficacy analysis.

c Referred to as SVR in the text and other tables and figures.

d W28VR, week 28 virologic response (HCV RNA was undetectable at week 28); RVR, rapid virologic response (HCV RNA was undetectable at week 4); SVR12, sustained virologic response, week 12 (HCV RNA was undetectable at posttreatment week 12); SVR24, sustained virologic response, week 24 (HCV RNA was undetectable at posttreatment week 24).

**FIG 5:**
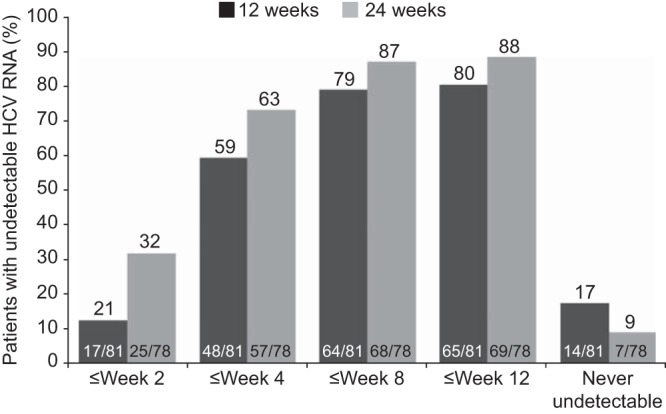
Proportion of patients with undetectable HCV RNA at different time points. Through week 12, more patients in the 24-week group than in the 12-week group achieved undetectable HCV RNA.

Virologic breakthrough occurred in 12% (10/81) and 9% (7/78) of patients in the 12- and 24-week groups, respectively (Table 3). Rates of relapse were similar across the two groups. Across both groups, approximately 2% of patients with undetectable levels of HCV RNA at week 2 relapsed. More patients achieved undetectable levels of HCV RNA up to week 12 in the 24-week group than in the 12-week group (Fig. 5).

**TABLE 3:** Treatment failure

Failure type	Failure rate (no. [%])by faldaprevir treatment duration					
	12 wk			24 wk		
	GT-1a (*n* = 40)	GT-1b (*n* = 39)	Total (*n* = 81^*a*^)	GT-1a (*n* = 29)	GT-1b (*n* = 48)	Total (*n* = 78^*b*^)
Virologic breakthrough^*c*^	6 (15.0)	3 (7.7)	10^*a*^ (12.3)	3 (10.3)	4 (8.3)	7 (9.0)
During FDV plus PegIFN/RBV	1 (2.5)	3 (7.7)	5^*a*^ (6.2)	3 (10.3)	4 (8.3)	7 (9.0)
During PegIFN/RBV only	5 (12.5)	0	5 (6.2)	0	0	0
Relapse^*d*^	4 (10.0)	1 (2.6)	5 (6.2)	2 (6.9)	4 (8.3)	6 (7.7)
Other^*e*^	9 (22.5)	3 (7.7)	12 (14.8)	3 (10.3)	4 (8.3)	7 (9.0)

a GT-1g, *n* = 1 (had virologic breakthrough); GT-6e, *n* = 1 (achieved SVR).

b GT-1g, *n* = 1 (achieved SVR).

c HCV RNA rebound of ≥1 log_10_ from nadir or rebound to ≥100 IU/ml if nadir HCV RNA was undetectable on treatment; confirmed in a second sample.

d Rebound posttreatment after HCV RNA was undetectable at the end of treatment; confirmed in second sample.

e Lost to follow-up, SVR result missing, other rebound (defined as confirmed rebound posttreatment when HCV RNA was detectable at the end of treatment), or any other SVR failure not described above.

The predominant emergent NS3/4A resistance-associated variants (RAVs) were R155K in 79% (15/19) of GT-1a virologic failures and D168V in 59% (10/17) of GT-1b virologic failures. All breakthroughs during faldaprevir/PegIFN/RBV treatment were associated with one of these RAVs, except one GT-1g R155K variant. Virologic breakthrough during PegIFN/RBV treatment occurred in five patients (Table 3): in four cases, this was associated with R155K GT-1a; no known RAVs were detectable in the other GT-1a case. Rarer NS3/4A RAVs detected during relapse included one each of R155S (GT-1a), D168E (GT-1a), and D168E (GT-1b).

### Safety.

The median faldaprevir treatment duration was 81 days for the 12-week group and 165 days for the 24-week group, with 93% (75/81) and 85% (67/79) of the 12- and 24-week groups, respectively, completing scheduled faldaprevir treatment. Median treatment duration for all HCV medication was 168 days for both treatment groups.

The open-label design of the study limits interpretation of the safety data. Most AEs were mild to moderate and were considered related to at least one of the study drugs. Six serious AEs occurred during the faldaprevir treatment phase, in three patients in each treatment group. Three serious AEs were considered related to treatment (anemia, neutropenia, and erythema); all resolved following treatment discontinuation, dose reduction, or without treatment modification. There were no deaths during the study. The most common AEs were gastrointestinal and were mostly mild. Most gastrointestinal AEs started during the first 8 weeks of treatment. In the 24-week group, one patient reported a severe rash (morbiliform). A second patient reported moderate rash during faldaprevir treatment, leading to discontinuation of all therapy. One patient in the 24-week group had a mild photosensitivity reaction during faldaprevir/PegIFN/RBV treatment. AEs reported during the faldaprevir phase are summarized in Table 4.

**TABLE 4:** AEs reported during 12 or 24 weeks of faldaprevir plus pegylated interferon α-2a and ribavirin treatment

AE	No. (%) of patients with AEs reported by faldaprevir treatment duration	
	12 wk (*n* = 80)	24 wk (*n* = 79)
Any	75 (94)	71 (90)
Serious^*a*^	3 (4)	3 (4)
Leading to discontinuation of faldaprevir	4^*b*^ (5)	5^*c*^ (6)
Anemia	8 (10)	14 (17.7)
Jaundice (total)	3 (3.8)	4 (5.1)
Mild	3 (3.8)	3 (3.8)
Moderate	0	1 (1.3)
Severe	0	0
Most common (>20% of patients in either arm)		
Gastrointestinal disorders		
Nausea	27 (34)	17 (22)
Skin disorders		
Pruritus	24 (30)	26 (33)
Rash	18 (23)	17 (22)
Dry skin	12 (15)	16 (20)
Nervous system disorders		
Headache	14 (18)	18 (23)
General disorders		
Asthenia	18 (23)	13 (17)
Fatigue	17 (21)	13 (17)

a Serious AEs in the 12-week group included anemia (two cases) and depression (one case). Serious AEs in the 24-week group included anemia, neutropenia, epilepsy, and erythema.

b One patient discontinued all treatment on day 8 due to prostate cancer diagnosed during screening. The three other patients who discontinued faldaprevir reported multiple AEs.

c One patient reported anemia, one reported rash, and the other three who discontinued faldaprevir reported multiple AEs.

Overall, 10 patients (12%) in the faldaprevir 12-week group and seven (9%) in the 24-week group reported severe AEs; these included six (8%) and seven (9%) patients, respectively, during faldaprevir therapy. Except for three cases (headache, hypothyroidism, and ulcerative colitis), all of the severe AEs resolved. One patient in each group experienced severe anemia (hemoglobin, <8.5 g/dl). Hemolytic anemia was reported in one patient during the faldaprevir 24-week treatment phase. All three patients with severe anemia achieved SVR.

Table 5 shows the incidence of marked laboratory changes on treatment. Similar proportions of patients experienced on-treatment laboratory abnormalities in the two faldaprevir treatment groups. Increases in total bilirubin levels, mostly owing to an increase in unconjugated bilirubin, were observed during faldaprevir treatment. Generally, aspartate aminotransferase (AST) and alanine aminotransferase (ALT) levels decreased from baseline during faldaprevir treatment. At study end, median laboratory values for hematologic parameters, bilirubin, and liver enzymes were within the reference ranges in both groups. Overall, four patients had a conjugated/total bilirubin ratio of >0.5 at any time during the trial. None of these patients had AST or ALT elevations greater than 3× ULN, none reported jaundice, and all returned to a ratio of ≤0.5 by study end. All four of these patients completed all treatments.

**TABLE 5:** Incidence of marked laboratory changes during 12 or 24 weeks of treatment with faldaprevir plus pegylated interferon α-2a and ribavirin

Laboratory parameter^*a*^	Value (no [%]) by faldaprevir treatment duration	
	12 wk (*n* = 81)	24 wk (*n* = 79)
Total bilirubin	79	78
1.1–1.5× ULN	25 (31.6)	22 (28.2)
1.6–2.5× ULN	18 (22.8)	24 (30.8)
2.6–5.0× ULN	6 (7.6)	6 (7.7)
>5.0× ULN	0	2^*b*^ (2.6)
ALT	79	78
1.25–2.5× ULN	37 (46.8)	38 (48.7)
2.6–5.0× ULN	15 (19.0)	17 (21.8)
5.1–10.0× ULN	3 (3.8)	4 (5.1)
>10.0× ULN	0	0
Hemoglobin	80	78
10.0–10.9 g/dl	18 (22.5)	16 (20.5)
9.0–9.9 g/dl	13 (16.3)	10 (12.8)
7.0–8.9 g/dl	3 (3.8)	4 (5.1)
<7.0 g/dl	0	0

a Standard reference ranges are the following: total bilirubin, 0.1 to 1 mg/dl; ALT, 0 to 35 U/liter; hemoglobin, 12.5 to 18 g/dl.

b Both patients had a conjugated/total bilirubin ratio of ≤0.5 and total bilirubin levels of 5.2× ULN and 6.6× ULN when the elevation occurred.

## DISCUSSION

The SILEN-C3 trial results show that a 12-week treatment duration of faldaprevir at 120 mg QD on a background of PegIFN/RBV achieves efficacy similar to that of 24 weeks of faldaprevir therapy for treatment-naive patients infected with HCV GT-1. High SVR rates of ≥67% and W28VR rates of ≥75% were obtained in a population which included approximately 12% of patients with liver cirrhosis.

The majority of patients (70% and 83% in the 12- and 24-week groups, respectively) met the response criterion (mRVR) for shortened PegIFN/RBV treatment and were eligible for 24 weeks of therapy. The mRVR rates were higher than the RVR rates, because RVR was defined as undetectable levels of HCV RNA at week 4, whereas mRVR included HCV RNA levels below the LLOQ at this time.

Despite receiving the same regimen until week 12, the 24-week group achieved higher rates of undetectable HCV RNA from week 2 onwards and had fewer patients who never achieved undetectable viral load compared to the 12-week group. Historically, for PegIFN/RBV-treated GT-1 patients, RVR at week 4 has been predictive of achieving SVR (17), and patients requiring a longer time to eliminate the virus from plasma may benefit from longer treatment. Previous studies of faldaprevir alone and combined with PegIFN/RBV (13) suggest that a reliable time point of SVR prediction is week 2 or earlier. In this study, SVR rates were similar for the 12- and 24-week groups in patients achieving undetectable levels of HCV RNA independent of the time to first negative HCV RNA result. This indicates that the lower response rates in the 12-week group compared with the 24-week group were due to an imbalance in baseline factors, including age, viral load of ≥800,000 IU/ml, and the proportion of patients who had HCV GT-1a. The small sample sizes and the absence of data on other host factors that predict the response to treatment, such as *IL28B* gene polymorphisms (18), precluded statistical analyses of factors determining the response.

A 3-day PegIFN/RBV lead-in period was included before the first dose of faldaprevir, reflecting mainstay treatment strategies at the time of trial onset. Following the commencement of this study, the phase 2 SILEN-C1 and -C2 studies reported that a strategy with no lead-in achieved SVR rates approximately 10% higher than those of lead-in regimens employing otherwise identical doses and durations (14, 19), although the reasons for these differences are unknown. Lead-in has not been incorporated into any phase 3 trial design for faldaprevir. This trial demonstrated the efficacy of the 120-mg dose of faldaprevir which has been used to inform phase 3 clinical trials whose results indicate that faldaprevir at 120 mg QD plus PegIFN/RBV without lead-in achieves SVR rates of 79% in treatment-naive patients (20).

Relapse and breakthrough rates for the 12- and 24-week groups were low and consistent with those observed for the same faldaprevir dose in SILEN-C1 (14). The predominance of the major NS3/4A resistance variant R155K in GT-1a and D168V in GT-1b has been observed in earlier studies, and these variants reduce the activity of faldaprevir *in vitro* (21); this resistance profile overlaps with that of other protease inhibitors (22).

The types and frequencies of AEs were similar in the 12- and 24-week groups despite the longer treatment with faldaprevir and PegIFN/RBV in the latter group. This may be because most AEs occurred in the first 12 weeks of therapy and/or because PegIFN/RBV contributed to the AEs (23). Safety and tolerability during faldaprevir treatment were consistent with SILEN-C1 (14) results, and no additional safety concerns were identified. Rash was reported at expected levels (22 to 23%), and only two patients discontinued treatment due to rash. Jaundice, caused by increases in unconjugated bilirubin, was generally mild, uncommon (4 to 5% of patients), and did not lead to treatment discontinuation. Faldaprevir inhibits UGT1A1 (the bilirubin-conjugating enzyme) and, to a lesser extent, the OATP1B1 and MRP2 transporters (24), similar to the HIV protease inhibitor atazanavir, which also causes transient benign elevations in unconjugated bilirubin, regarded as a cosmetic problem rather than a toxicity (25).

Although no direct comparison was made, the incidences of anemia and drug discontinuations due to AEs observed with faldaprevir were lower than those that have been observed in studies with telaprevir and boceprevir (5, 6). This suggests that faldaprevir at 120 mg QD plus PegIFN/RBV enables more treatment-naive patients with HCV GT-1 to receive a 24-week course of treatment. Simeprevir QD has also achieved increased SVR rates compared to those of PegIFN/RBV treatment alone (26). Unlike faldaprevir, simeprevir does not inhibit UGT1A1 but inhibits OATPs and MRP2 (27), causing increases in conjugated and unconjugated bilirubin.

Major limitations of the current study include the open-label design, the absence of a PegIFN/RBV control arm, and the lack of *IL28B* testing. Due to the open-label design, safety and tolerability comparisons should be made with caution, but the efficacy endpoints are objective and should not be affected by the unblinded treatment duration. Within the phase 2 program for faldaprevir, SILEN-C3 was designed to investigate the efficacy and safety of response-guided faldaprevir at 120 mg for 12 weeks versus 24 weeks, while the randomized, double-blind SILEN-C1 study compared faldaprevir plus PegIFN/RBV treatment to PegIFN/RBV alone (14). At the time of initiation of SILEN-C3, the impact of *IL28B* genotype on response was not known.

SILEN-C3 showed that addition of faldaprevir at 120 mg QD to PegIFN/RBV enables most treatment-naive HCV GT-1 patients to receive a total of 24 weeks of therapy, achieves high SVR rates, and is well tolerated. These results support the use of the 120-mg daily dose of faldaprevir currently being investigated in phase 3 clinical trials.
